# Tuning Polymer/TiO_2_ Nanocomposites Morphology by In Situ Non-Hydrolytic Sol-Gel Syntheses in Viscous Polymer Medium: Influence of the Polymer Nature and Oxygen Donor

**DOI:** 10.3390/polym14112273

**Published:** 2022-06-02

**Authors:** Manon Besançon, Yanhui Wang, Johan G. Alauzun, Hubert Mutin, Eliane Espuche, Véronique Bounor-Legaré

**Affiliations:** 1 Ingénierie des Matériaux Polymères, UMR CNRS 5223, CNRS, Université de Lyon, Université Claude Bernard Lyon 1, INSA de Lyon, Université Jean Monnet, F-69622 Lyon, France; manonbesancon@laposte.net (M.B.); eliane.espuche@univ-lyon1.fr (E.E.); 2 ICGM, Université de Montpellier, CNRS UMR 5253, ENSCM, F-34296 Montpellier, France; wangyh1987@126.com (Y.W.); johan.alauzun@umontpellier.fr (J.G.A.); hubert.mutin@umontpellier.fr (H.M.)

**Keywords:** non hydrolytic sol-gel, nanocomposites, polymer processing, PP, PS

## Abstract

Herein, we reported the synthesis of TiO_2_ through different non-hydrolytic sol-gel (NHSG) routes in viscous polymer media. For the first time, the influence of the polymer nature (Polystyrene (PS) or Polypropylene (PP)) on the morphology of synthesized inorganic domains was investigated. The non-hydrolytic sol-gel reactions between titanium isopropoxide Ti(O^i^Pr)_4_ and acetic anhydride in molten polypropylene lead to the formation of microfillers with a mean diameter of about 1 μm, while the same synthesis carried out in viscous polystyrene lead to the formation of nanofillers with diameter lower than 10 nm forming aggregates of approximately 200 nm. We have also investigated the influence of the oxygen donor nature on the morphology of synthesized fillers using aromatic oxygen donors in a polystyrene matrix. The use of benzoic anhydride or acetophenone as oxygen donors with Ti(O^i^Pr)_4_ in viscous polystyrene lead to respectively platelet-like morphology or aggregated nanofillers. We demonstrated that the affinity between polymer, reactants, and/or by-products had an influence on the morphology and the size of in situ synthesized TiO_2_ fillers. These results evidenced for the first time the possibility to control and to tune the morphology of in situ grown inorganic objects through the NHSG process by the appropriate choice of solvent, here a viscous polymer medium, and reactants.

## 1. Introduction

Polystyrene (PS) and polyolefins are commonly used due to their mechanical robustness, availability, processability, and low cost. Besides their use as pure matrices, they are often combined with inorganic fillers to reach specific properties such as barrier, mechanical, conductive properties, etc. These properties are highly dependent on the nanofillers dispersion [1,2,3]. However, dispersing hydrophilic fillers such as oxides in hydrophobic matrices remains challenging. For example, Kumari et al. [4] studied the dispersion of TiO_2_ nanofillers in a PS matrix by a solvent cast method. A total of 5 wt% of TiO_2_ anatase nanofillers of approximately 20 nm in diameter synthesized by a hydrolytic sol-gel process from titanium isopropoxide were agglomerated (about 100 μm) with a heterogeneous distribution in the PS matrices.

Thus, in order to reach good nanofiller dispersion, a pre- or post-modification of fillers and/or polymer matrix is often required. Zan et al. [5] have elaborated PS/TiO_2_ nanocomposites by grafting polystyrene chains at the surface of anatase TiO_2_ nanofillers of 70–100 nm in diameter. Modified fillers were then dispersed in styrene monomer with an initiator (also AIBN) and the polymerization was performed at 105 °C for 15 h. These nanocomposites presented a better filler dispersion state than PS/TiO_2_ nanocomposites obtained from pristine TiO_2_ with a mean filler diameter between 100 and 300 nm (in comparison with size between 500 and 600 nm for pristine TiO_2_). The addition of a compatibilizer to promote the dispersion of TiO_2_ nanofillers is also a common strategy. For example, the polypropylene grafted by maleic anhydride (PP-g-MA) was employed by Womble et al. [3] to improve the dispersion of TiO_2_ nanofillers in a polypropylene (PP) matrix. PP/TiO_2_ nanocomposites with 15vol% of fillers (with a diameter size of around 30 nm) were prepared with or without adding PP-g-MA through a suspension in hot toluene. After mixing and evaporation of the solvent, a more homogeneous distribution of nanofillers was observed in presence of PP-g-MA which reduced the nanofillers surface energy, avoiding their aggregation.

Besides, there is more and more concern about the potential toxicity of nanofillers [6]. Thus, novel ways of composites elaboration have been proposed, in particular, the in situ synthesis of inorganic fillers into a molten polymeric matrix by sol-gel process [7,8,9,10,11]. The sol-gel process is particularly suitable for the synthesis of inorganic domains in organic polymer given the mild conditions of synthesis. Indeed, the sol-gel chemistry is a method of “chimie douce” [12] which involves a temperature range compatible with the classical melt process of most commodity polymers. To illustrate, in a previous work, we have already described the synthesis of a TiO_2_ filler into molten polypropylene via a non-hydrolytic sol-gel route [13]. PP/TiO_2_ composites were prepared in an extruder at 200–240 °C. Two oxygen donors were used in order to evaluate their role on the TiO_2_ filler dispersion in the polymer. The use of titanium isopropoxide with acetic anhydride led to the formation of filler particles with a mean size of 1 μm. The dispersion of the TiO_2_ filler was slightly enhanced by replacing acetic anhydride with hexanoic anhydride: the mean size of fillers was then around 700 nm.

Besides the nature of the inorganic precursors’ impact, it is well-known that the nature of the solvent can influence sol-gel reactions. For example, Hu et al. [14] demonstrated, by comparing ethanol, ethanol mixed with isopropyl alcohol, and ethanol with 2-ethoxyethanol, that the addition of isopropyl alcohol or 2-ethoxyethanol promoted the crystallization of TiO_2_ obtained by the hydrolysis-condensation of titanium isopropoxide. It resulted also in an increase in the surface area. Jiang et al. [15] synthesized zirconium silicate nanofillers from the non-hydrolytic sol-gel reaction between zirconium tetrachloride and tetraethoxysilane and demonstrated that the use of N,N-dimethylformamide (DMF) led to the agglomeration of nanofillers, whereas the use of dichloromethane allowed obtaining well-dispersed nanofillers of 30 nm in diameter. They also demonstrated that the use of ethanol as solvent led to the formation of smaller fillers (mean fillers size of 20–30 nm) than using propanol or isopropanol (mean fillers size larger than 50 nm forming agglomerates). Actually, as the reaction temperature was lower for fillers synthesized in ethanol, a lower condensation degree was reached which can explain the smallest fillers in this case. More recently, Wang et al. [16] reported the synthesis of mesoporous TiO_2_ by three different non-hydrolytic sol-gel (NHSG) routes: the common ether route between titanium chloride and diisopropyl ether with alkyl halide elimination and two original routes involving the use of titanium isopropoxide as titanium dioxide precursor and acetophenone or benzoic anhydride as oxygen donor. Syntheses were carried out in three different liquid media: without solvent (the oxygen donor acts as solvent), in toluene and in squalane. The ether route between titanium chloride and diisopropyl ether in toluene led to the formation of well-dispersed rod-shape nanoparticles, whereas the same synthesis performed in squalane or without solvent formed aggregates of few micrometers. The TiO_2_ primary nanoparticles synthesized from acetophenone in squalane formed spherical secondary aggregates (1–15 μm), whereas in toluene, nanoparticles formed large aggregates but without specific morphology. Conversely, benzoic anhydride as oxygen donor led to the formation of spherical aggregates in the case of the use of toluene and shapeless aggregates with the squalane as solvent.

In the case of polymer nanocomposite elaboration by in situ synthesis of the inorganic filler through a molten process, the viscous polymer will act as a solvent. Thus, the choice of the polymer matrix could therefore have an influence on the nanofiller shape and dispersion, as that can be classically observed in sol-gel chemistry. The aim of this study was first to evaluate the influence of the polymer matrix nature on the shape and the crystallinity of inorganic fillers. Within this context, the in situ non-hydrolytic sol-gel synthesis of TiO_2_ from acetic anhydride and titanium isopropoxide in two different media, molten polypropylene and viscous polystyrene was investigated. Secondly, specifically for the polystyrene medium, the influence of benzoic anhydride and acetophenone, as two aromatic oxygen donors, on the morphology of the TiO_2_-PS based nanocomposites was investigated and discussed in terms of reactants/matrix and fillers/matrix interactions during the synthesis.

## 2. Experimental Part

### 2.1. Materials

The two polymer matrices used were the polypropylene (PP) “Moplen HP500N” from LyonDellBasell (melt flow index (MFI) of 12 g.10 min^−1^ at 230 °C/2.16 kg) and the polystyrene (PS) “Lacqrene 1240” from Total (MFI of 2.6 g.10 min^−1^ at 200 °C/5 kg). TiO_2_ Aeroxide P25 nanoparticles with primary particle size of about 21 nm (purchased from Sigma Aldrich) were used as references. Titanium (IV) isopropoxide 97% purchased from ABCR was used as titanium dioxide precursor. Acetic anhydride ≥99% was purchased from Carlo Erba. Benzoic anhydride ≥95% and acetophenone 99% were purchased from Sigma Aldrich (France). All chemicals were used as received. Several properties of the reactants which were of interest for the synthesis protocol considered in this work are depicted in Table 1.

### 2.2. Nanocomposites Preparation

Titanium isopropoxide and the oxygen donor (acetic anhydride (Ac_2_O), benzoic anhydride (BzA) or acetophenone (Aph) respectively) in stoichiometric proportions (2 eq of oxygen donor relative to titanium isopropoxide) were added to the molten polypropylene or viscous polystyrene in a Haake^®^ (Germany) internal mixer equipped with two rotors R600 running in a counter rotating way. For the use of acetic anhydride as oxygen donor, reactants (Ti(O^i^Pr)_4_ and anhydride) were pre-mixed as previously described [13]. The rotor speed was fixed at 50 rpm. Amounts of reactants were defined in order to achieve a TiO_2_ concentration of 5wt%. The different steps of the nanocomposite preparation were as follows: first, the polymer was incorporated in the mixing chamber at 200 °C. After three minutes, the titanium dioxide precursor and the selected oxygen donor were added to the polymer medium in the mixing chamber. After 5 min of mixing at 200 °C, the temperature was gradually increased from 200 °C up to240 °C (Heating rate = 10 °C min^−1^). Finally, 15 min after the introduction of reactants, rotors were stopped and the nanocomposite recovered. For comparison, PP/TiO_2_ and PS/TiO_2_ nanocomposite references with 5wt% of a commercial TiO_2_ filler (Aeroxide P25) were prepared using the same mixing conditions. Neat PP and PS references were also prepared in the internal mixer with the same experimental conditions. Table 2 summarizes all samples prepared and reports the by-products expected to be formed from the synthesis reaction.

Note that as the boiling point of isopropyl acetate is lower than the reaction temperature, it should then be eliminated during the synthesis. Residual by-products from reactions with benzoic anhydride and acetophenone were extracted from PS-based nanocomposites by immersion of the nanocomposites (grounded into a powder) in glacial acetic acid at room temperature and under stirring for 24 h. In situ formed nanofillers were extracted from PS-based nanocomposites by dissolution in toluene at room temperature and centrifugation (three times).

### 2.3. Nanocomposites Characterization

Thermogravimetric analyses (TGA) were performed on polymers and nanocomposites with a TA Q600 apparatus (TA Instruments, New Castle, DE, USA). Analyses were carried out under helium atmosphere at the flow rate of 60 mL.min^−1^. Samples were treated from room temperature up to 600 °C at 10 °C.min^−1^.

Residual reactants and by-products were identified by thermal desorption analysis (TDA, Turbo Matrix 350 instrument from Perkin Elmer, France), gas chromatography (GC, Clarus 680 from Perkin Elmer) and mass spectroscopy (MS Clarus SQ 8T from Perkin Elmer) coupling techniques. TDA analyses were realized under a helium flow rate of 1 mL min^−1^. Nanocomposite samples were treated at 200 °C for 10 min. The released volatiles were transferred to a gas chromatograph Clarus 680 of Perkin Elmer and a mass spectrometer Clarus SQ 8T of Perkin Elmer.

The crystalline structure of nanocomposites was analyzed by X-ray diffraction (XRD) on composites ground to fine powder using Cu Kα radiation (*λ* = 1.54 Å). Data were collected in 2 Theta from 1–6° thanks to a diffractometer PANalytical X’Pert Pro Spinner (Step size 0.05°, Time/Step120s) and from 5–50° thanks to a diffractometer Bruker D8-Advance (Step size 0.02°, Time/step 1.2 s), respectively.

Fourier Transform Infrared (FTIR) spectroscopy in attenuated total reflection mode of nanocomposites was recorded with a Nicolet iS-10 Fourier transform infrared spectrometer of Thermo Scientific in the wavelength range of 650–4000 cm^−1^. The number of scans was 64 and the resolution was 4 cm^−1^.

The Scanning Electron Microscopy (SEM) observations of nanocomposites were realized with a microscope FEI Quanta 250 FEG microscope (Voltage = 10 kV). Backscattered electron mode allowed to enhance the contrast between polymer matrix and titanium dioxide fillers. Observed sections were prepared by ultra-microtomy at −110 °C for PP-based systems and at room temperature for PS-based systems. Fillers Feret’s diameters were determined using the ImageJ software from SEM images. The Transmission Electron Microscopy (TEM) observations were performed with a Philips CM120 microscope using an accelerate voltage of 80 kV. Ultrathin sections were made using the same equipment as for SEM sample preparation. The thickness of sections is about 80 nm.

The analysis by X-ray photoelectron spectroscopy (XPS, PHI Quantera SXM spectrometer) was realized with a non-monochromatized AlKα source. A 45° take-off angle between the sample and the analyzer was chosen. Multipak (PHI) software allowed reaching the spectra deconvolution. Two energies were respectively used: 280 eV for sounding spectra and 140 eV for high resolution spectra.

## 3. Results and Discussion

In order to demonstrate the possibility to control the shape of the TiO_2_ synthesized by NHSG in a viscous polymer, two ways were deeply scrutinized: (i) First, the influence of the polymer matrix nature on the filler growth and nanocomposite morphology was investigated; (ii) Then the synthesis of TiO_2_ fillers from two different aromatic oxygen donors in polystyrene was studied, in particular, the effect of oxygen donor on the filler shape was discussed.

### 3.1. Acetic Anhydride Route: Influence of the Polymer Matrix Nature

The in situ non-hydrolytic sol-gel synthesis of TiO_2_ from Ti(O^i^Pr)_4_ and Ac_2_O was already described in our previous work [13]. The affinity between acetic anhydride and polypropylene or polystyrene matrices was investigated by the determination of their solubility parameters δ by the Fedors’ method, which requires only the knowledge of the compound structural formula [17]. Calculated values of δ are given in Table 3.

Hildebrand and Hansen solubility parameters of the polypropylene were estimated between 15.5 and 17.5 (MPa)^1/2^ with a preferred value of 16.5 (MPa)^1/2^ [18] close to the calculated value of 16.0 (MPa)^1/2^ by the Fedors’ method. For polystyrene, Hildebrand and Hansen solubility parameters are between 18.2 and 20.2 (MPa)^1/2^ [18], thus slightly lower than the value calculated from Fedor’s method. As the acetic anhydride has a calculated solubility parameter of 19.8 (MPa)^1/2^, it may have more affinity with polystyrene (Δδ = 1.3 (MPa)^1/2^) than with polypropylene (Δδ = 3.8 (MPa)^1/2^). Based on this parameter, a better dispersion should be consequently expected for the PS-based composite in situ synthesized from acetic anhydride than for the PP-based composite.

**Figure 1:**
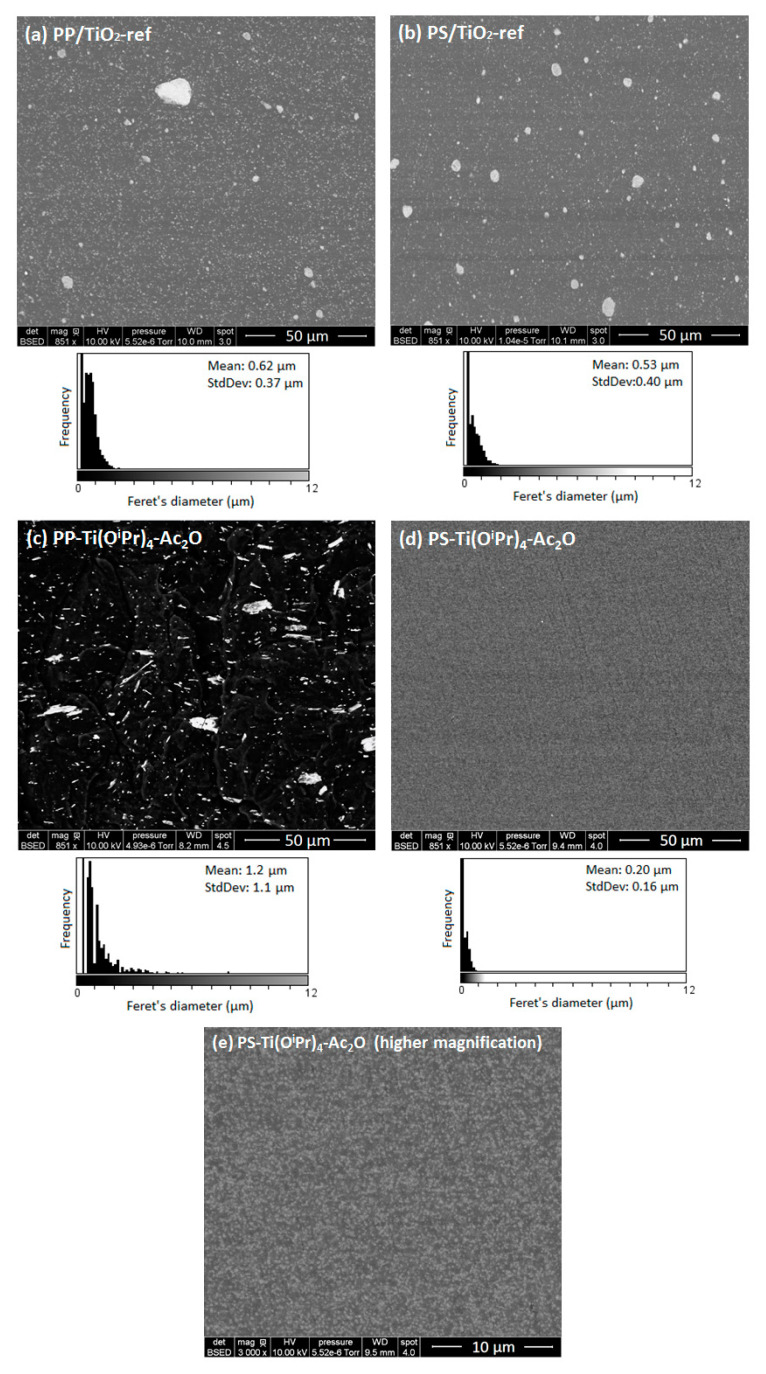
SEM images and Feret’s diameter distribution of filler particles in (**a**) PP/TiO_2_ nanocomposites prepared by incorporation of Aeroxide P25 TiO_2_ (PP/TiO_2_-ref), (**b**) PS/TiO_2_ nanocomposites prepared by incorporation of Aeroxide P25 TiO_2_ (PS/TiO_2_-ref), (**c**) PP/TiO_2_ nanocomposites prepared by in situ NHSG synthesis from Ti(O^i^Pr)_4_ and Ac_2_O (PP-Ti(O^i^Pr)_4_-Ac_2_O), (**d**) PS/TiO_2_ nanocomposites prepared by in situ NHSG synthesis from Ti(O^i^Pr)_4_ and Ac_2_O (PS-Ti(O^i^Pr)_4_-Ac_2_O), (**e**) PS-Ti(O^i^Pr)_4_-Ac_2_O nanocomposite at higher magnification.

To evaluate this effect, the morphology of the in situ formed nanocomposites was investigated by electron microscopy (Figure 1) and compared to the morphology of reference nanocomposites (e.g., nanocomposites prepared using a commercial TiO_2_ filler). Concerning the morphologies of the two reference nanocomposites (PP/TiO_2_-ref and PS/TiO_2_-ref), we can observe that the TiO_2_ particles distributed in both composites are mainly with a sub-micronic diameter with some large, micron-sized aggregates. The mean Feret’s diameter of observed aggregates is about 0.62 μm for PP/TiO_2_-ref sample and 0.53 μm for PS/TiO_2_-ref sample. Concerning the nanocomposites elaborated by the in situ NHSG synthesis of TiO_2_ from the reaction between Ti(O^i^Pr)_4_ and Ac_2_O, morphologies are very different. For the PP-Ti(O^i^Pr)_4_-Ac_2_O composite, a wide distribution of fillers size is observed. The mean Feret’s diameter is about 1.18 µm but some filler particles are larger than 10 μm. Consequently, this in situ NHSG synthesis of TiO_2_ in molten polypropylene does not lead to a better dispersion than the direct introduction of nanofillers by “top-down” approach.

**Figure 2:**
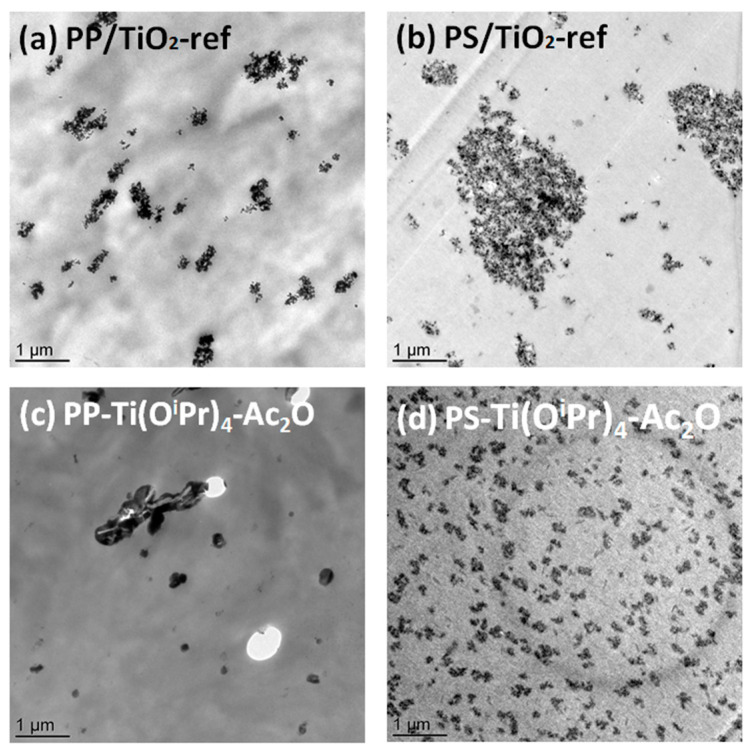
TEM images of (**a**) PP/TiO_2_ nanocomposites prepared by incorporation of Aeroxide P25 TiO_2_ (PP/TiO_2_-ref), (**b**) PS/TiO_2_ nanocomposites prepared by incorporation of Aeroxide P25 TiO_2_ (PS/TiO_2_-ref), (**c**) PP/TiO_2_ nanocomposites prepared by in situ NHSG synthesis from Ti(O^i^Pr)_4_ and Ac_2_O (PP-Ti(O^i^Pr)_4_-Ac_2_O) and (**d**) PS/TiO_2_ nanocomposites prepared by in situ NHSG synthesis from Ti(O^i^Pr)_4_ and Ac_2_O (PS-Ti(O^i^Pr)_4_-Ac_2_O).

For the PS-Ti(O^i^Pr)_4_-Ac_2_O sample, the filler particles are homogeneously dispersed in the polymer with a mean diameter of 200 nm. The size distribution is narrow in comparison with the PS/TiO_2_-ref sample. These first results are consistent with the prediction conducted from the determination of the solubility parameters (Table 3) where a better dispersion of the filler was expected in the PS matrix. To go further in the morphology analysis, TEM analysis was performed. The transmission electron micrographs presented in Figure 2 show that the dispersed domains observed at the SEM scale for PP/TiO_2_-ref, PS/TiO_2_-ref and PS-Ti(O^i^Pr)_4_-Ac_2_O samples are in fact composed of aggregates of TiO_2_ nanoparticles. In situ synthesized TiO_2_ primary nanoparticles in PS-Ti(O^i^Pr)_4_-Ac_2_O nanocomposite have diameters below 10 nm, smaller than Aeroxide P25 TiO_2_ primary particles which have an average diameter of 21 nm. These nanoparticles are assembled to form well-dispersed aggregates of around 200 nm. For the PP-Ti(O^i^Pr)_4_-Ac_2_O sample observed by TEM (Figure 2c), the morphology is more complex and a wide range of particle sizes is observed. Small fillers seem rather spherical whereas big filler particles have a rod-shaped morphology. There are also some holes in the observed section, induced by large filler particles torn from the polypropylene matrix indicating a poor adhesion between the TiO_2_ filler and the PP matrix.

These observations demonstrated that NHSG chemistry allowed the in situ formation of TiO_2_ filler in both PP and PS matrices, with a morphology that is highly dependent on the polymer structure. The determination of the solubility parameter gave a first clue to explain this difference in morphologies. However as detailed in the introduction, other parameters can contribute to this difference in morphology such as, for example, the condensation degree.

**Figure 3:**
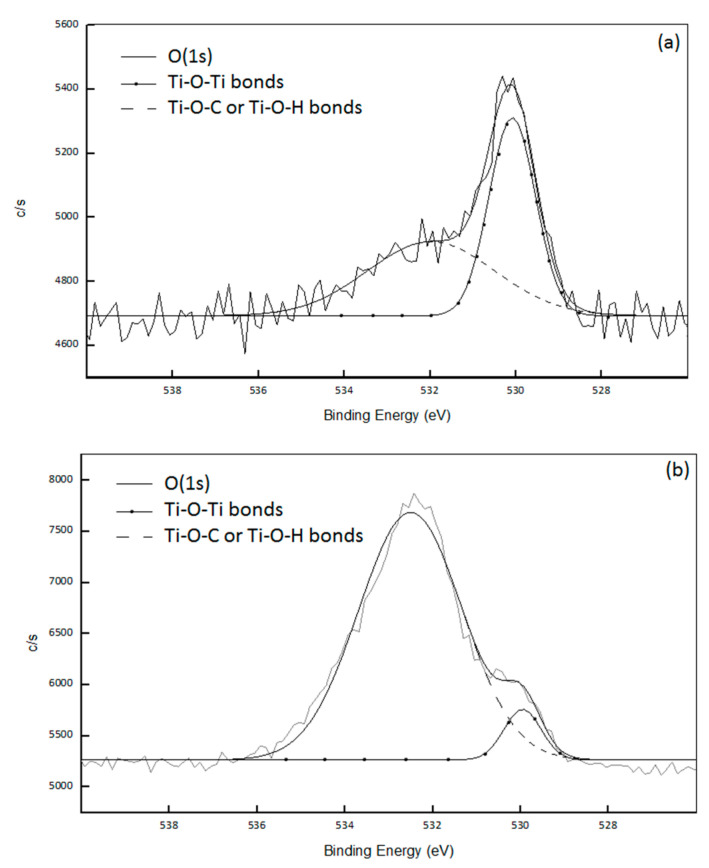
Deconvolution of the XPS O(1s) signal of (**a**) the PP-Ti(O^i^Pr)_4_-Ac_2_O sample and (**b**) the PS-Ti(O^i^Pr)_4_-Ac_2_O sample.

To estimate the condensation degree of TiO_2_ synthesized in situ by non-hydrolytic sol-gel reactions, XPS measurements were performed on PP-Ti(O^i^Pr)_4_-Ac_2_O and PS-Ti(O^i^Pr)_4_-Ac_2_O composites. The atomic concentrations are the following: 98.9% C, 0.8% O and 0.3% Ti for the PP-Ti(O^i^Pr)_4_-Ac_2_O composite and 95.0% C, 3.9% O and 0.2% Ti for the PS-Ti(O^i^Pr)_4_-Ac_2_O composite. According to the Ti/O ratio, a higher condensation degree is expected for PP-Ti(O^i^Pr)_4_-Ac_2_O composite. Actually, the XPS analysis provides information about the condensation degree of synthesized titanium dioxide through the quantification of –Ti–O–Ti–, –Ti–O–C– or –Ti–O–H bonds respectively. Deconvolution of the O(1s) signal for the two composites (Figure 3) allowed to determine the different environments of oxygen atoms in the created nanofillers: the signal at 529.9 eV corresponded to –Ti–O–Ti– and the one at 531.8 eV to –Ti–O–H or –Ti–O–C [19,20,21,22,23,24]. For PP-Ti(O^i^Pr)_4_-Ac_2_O sample, 65% of oxygen atoms were in a –Ti–O–Ti environment and 35% of oxygen atoms were in a –Ti–O–C or –Ti–O–H environment. The mean formula of the Ti domain is therefore Ti(O)_1.58_(OR)_0.84_ which implies a condensation degree of 79%. The condensation degree calculation was already detailed in a previous publication [13] For the PS-Ti(O^i^Pr)_4_-Ac_2_O nanocomposite, the deconvolution of the O(1s) signal (Figure 3b) allowed to determine that 11% of O atoms were involved in –Ti–O–Ti bonds and 89% were involved in -Ti–O–C or –Ti–O–H bonds, giving a condensation degree of 20%.

This difference in condensation degree: 79% for the PP-Ti(O^i^Pr)_4_-Ac_2_O sample and 20% for PS-Ti(O^i^Pr)_4_-Ac_2_O sample explained also the finest size of the filler in the PS matrix as also pointed out by Jiang et al. [15]. The large difference in viscosity (higher for the PS compared to PP one) can contribute to the decrease of the condensation degree by limiting the probability of reactions between chemical species.

However, other contributions such as the initial miscibility of the reactants and also the phase separation scenario depending on the NHSG reactions advancement may be involved.

**Figure 4:**
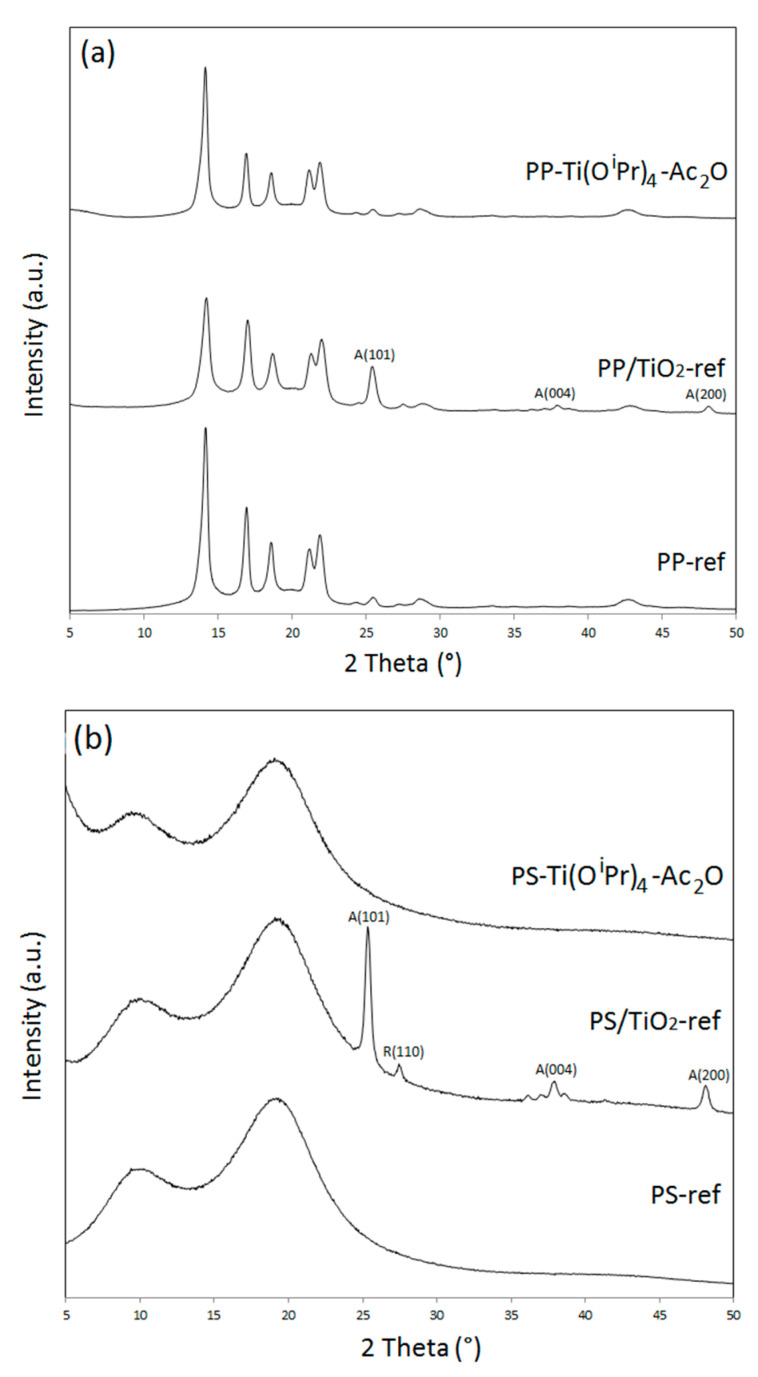
X-ray diffractograms at wide angles (5–50°) of (**a**) PP-ref and PP-based nanocomposites and (**b**) PS-ref and PS-based nanocomposites.

The structure of the filler was then studied through XRD analysis. The powder XRD patterns of the neat polymers and their respective polymer/TiO_2_ nanocomposites at wide angles, i.e., between 5 and 50°, are displayed in Figure 4. Polypropylene is a semi-crystalline polymer and the diffractogram of PP-ref corresponds to the α-monoclinic form of isotactic polypropylene [25]. On the PP/TiO_2_-ref diffractogram in Figure 4a, three new signals at 25.3°, 37.8°, and 47.9° are distinctly observed. These three signals are respectively related to the (101), (004), and (200) crystal planes of anatase TiO_2_ [26]. However, in the PP-Ti(O^i^Pr)_4_-Ac_2_O composite pattern, the absence of signals other than those of the polypropylene ones evidenced that the in situ synthesized TiO_2_ was in an amorphous state. These observations are consistent with previous results obtained for in situ hydrolytic sol-gel synthesis of TiO_2_ in molten polypropylene [9,19] and also with our first study dedicated to the in situ NHSG synthesis of TiO_2_ in polypropylene [13].

A similar behavior is observed for the PS-based composites (Figure 4b). The in situ synthesized TiO_2_ was in an amorphous state in the PS-Ti(O^i^Pr)_4_-Ac_2_O nanocomposite as in the case of PP-Ti(O^i^Pr)_4_-Ac_2_O. As polystyrene is an amorphous polymer, its X-ray diffractogram features two broad signals at 10° and 20°. In the PS/TiO_2_-ref sample, diffraction peaks due to the presence of nano-crystalline TiO_2_ are therefore easily distinguished. The same three peaks corresponding to anatase structure as previously observed are present, as well as a weak peak at 27.4° corresponding to the (110) crystal plane of rutile form [26]. In the PP/TiO_2_-ref diffractogram, this peak was not observed due to the overlap with the crystalline PP peaks. Actually, the commercial TiO_2_ filler used includes about 80% of anatase phase and 20% of rutile phase [27].

The amorphous nature of the filler created by NHSG reactions is consistent with our previous results [7,9,13,19] and the present processing conditions: short reactions times and viscous reactional medium.

**Figure 5:**
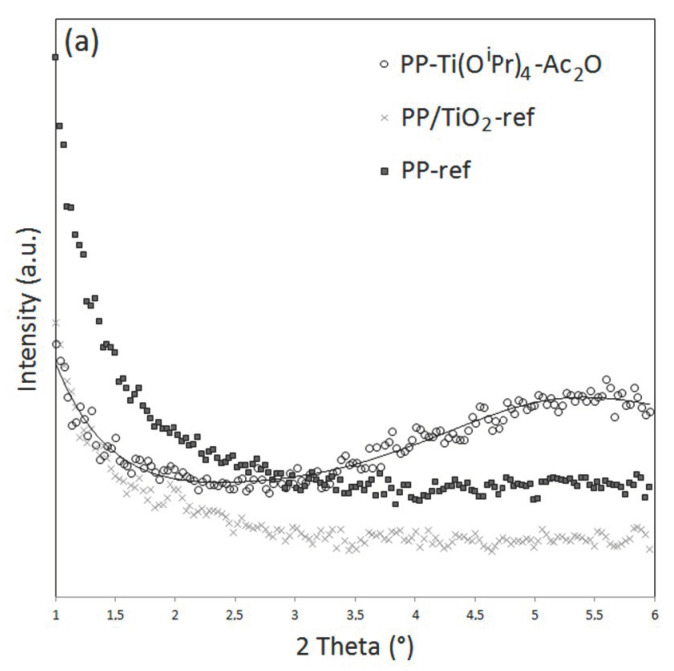

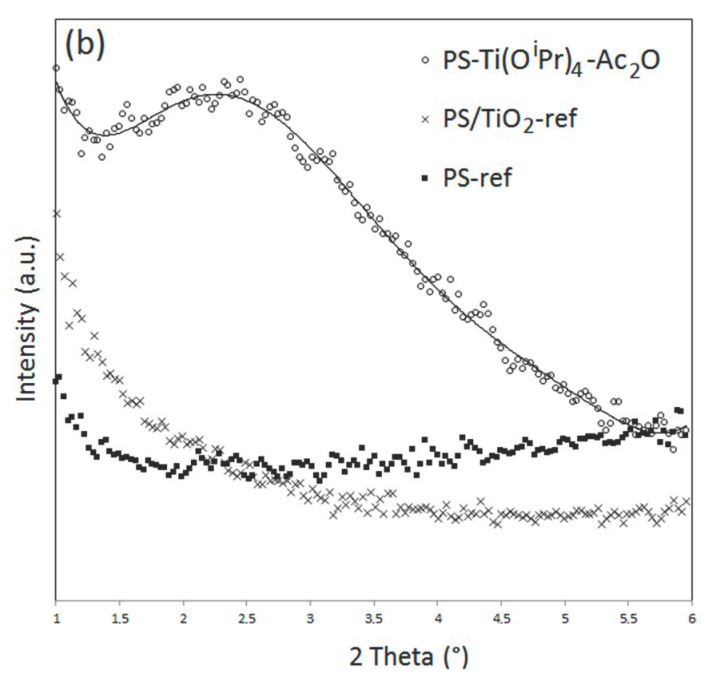
X-ray diffractograms at low angles (1–6°) of (**a**) PP-ref and PP-based nanocomposites and (**b**) PS-ref and PS-based nanocomposites.

In addition, a rise in signal intensity at a low angle is observed on the diffractograms of PS-Ti(O^i^Pr)_4_-Ac_2_O. In order to investigate the material’s behavior at a low angle, XRD measurements were performed in the 2Ɵ range between 1 and 6° (Figure 5). These analyses confirmed the presence of a broad intense signal centered at 2.2° for the PS-Ti(O^i^Pr)_4_-Ac_2_O nanocomposite but not for the PS and PS/TiO_2_ references. For PP-Ti(O^i^Pr)_4_-Ac_2_O sample, a broad less intense signal centered at 5.5° can be also observed for the composites obtained in situ. This signal has already been observed in other studies concerned with the in situ generation of TiO_2_ nanofillers [19,28] and related to a nanofiller dispersion state in the polymer matrix. Actually, Wu [28] by studying the XRD pattern of PCL/TiO_2_ and PCL-g-AA/TiO_2_ elaborated by the in situ synthesis of TiO_2_ fillers into the molten polymer evidenced a signal shift from 2.9° for PCL/TiO_2_ down to 2.6° for PCL-g-AA/TiO_2_. The authors explained the shift of angle by a better nanofiller dispersion state in the PCL-g-AA/TiO_2_ nanocomposite thanks to the grafted polymer. Based on these observations, our results could corroborate the most homogeneous dispersion observed by electron microscopy analysis for the PS-based system. Note that the signal at a very low angle is that of the direct beam.

All these data confirmed that the in situ synthesis of TiO_2_ filler by NHSG led to finer and more homogeneous dispersed fillers in a viscous PS compared to the ones created in a PP matrix due to the difference of affinity between the inorganic precursor and the matrix, the polymer viscosity, and the condensation degree. These observations are very interesting and agree with that is observed classically for NHSG carried out in liquid media. It confirmed that our viscous polymer can be considered as a specific “solvent” medium impacting the final morphology of the nanocomposites.

Finally, the thermal stability of the nanocomposites was evaluated. TGA thermograms in Figure S1 evidenced the absence of mass loss before 300 °C for both PP-Ti(O^i^Pr)_4_-Ac_2_O and PS-Ti(O^i^Pr)_4_-Ac_2_O, confirming the complete evaporation of by-products during the synthesis, as previously postulated. A slight mass loss between 310 and 360 °C was observed in PP-Ti(O^i^Pr)_4_-Ac_2_O and PS-Ti(O^i^Pr)_4_-Ac_2_O samples thermograms, which could correspond to by-products elimination throughout the residual condensation reactions occurring during the TGA analysis. 

Overlay of PS-ref, PS/TiO_2_-ref and PS-Ti(O^i^Pr)_4_-Ac_2_O thermograms (Figure S1b) evidenced a modification of the degradation temperature range with the introduction of TiO_2_ in the PS matrix. Actually, the polystyrene degradation for the neat polymer occurs between 350 and 440 °C, whereas the polystyrene matrix degradation for the two PS/TiO_2_ composites starts at 380 °C. The presence of TiO_2_ fillers leads to an improvement of the thermal stability of the PS matrix. This behavior has already been reported for PS/TiO_2_ nanocomposites [29,30,31]. According to the authors, the presence of TiO_2_ nanofillers results in reduced mobility of the polymer chains and therefore enhances the energy needed for polystyrene chain breakage. A slight increase in polypropylene degradation temperature was also observed for PP-Ti(O^i^Pr)_4_-Ac_2_O in comparison with the PP-ref sample in Figure S1a. Actually, PP degradation starts at 385 °C for the PP-Ti(O^i^Pr)_4_-Ac_2_O composite whereas for neat PP, the mass loss starts at a lower temperature around 350 °C. It is noteworthy that it remains about 5wt% of inorganic moieties after 500 °C in each nanocomposite, as expected.

### 3.2. Impact of Aromatic Oxygen Donors Nature with Polystyrene Matrix

Based on these previous results and the literature analysis [13,16], it appears interesting, complementary to the influence of the polymer nature, to study the impact of other oxygen donors on the nanofiller formation and resulting nanocomposite morphology. In particular, oxygen donors with aromatic groups should be suitable to promote interactions between TiO_2_ fillers and polystyrene matrix. For that purpose, two aromatic molecules were selected as oxygen donors to react with Ti(O^i^Pr)_4_: benzoic anhydride and acetophenone. To estimate the affinity of these two molecules with the polystyrene matrix, solubility parameters were calculated according to Fedor’s method [17]. The theoretical values for benzoic anhydride and acetophenone are respectively 22.9 and 21.1 (MPa)^1/2^, thus close to the value of polystyrene (δ_PS_ = 21.1 (MPa)^1/2^) in particular for acetophenone.

First, the system with benzoic anhydride was discussed by successively evaluating the reaction mechanism and advancement and more specifically the condensation degree followed by the morphology analysis.

**Figure 6:**
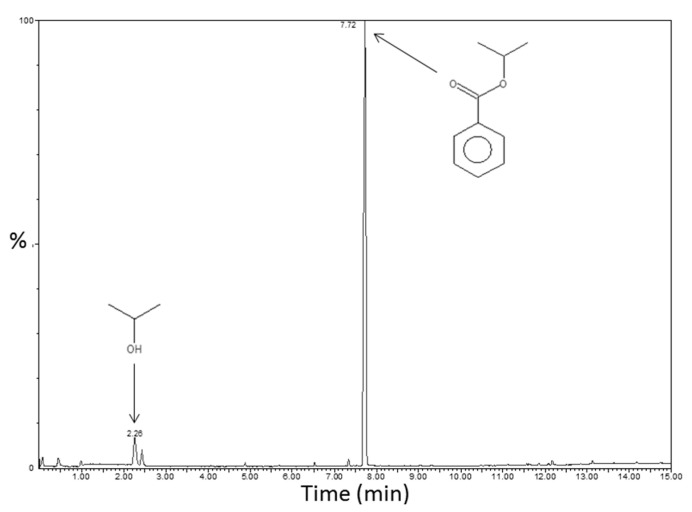
Chromatogram of the PS-Ti(O^i^Pr)_4_-BzA nanocomposite with signals identification obtained by TDA-GC-MS coupling analyses (200 °C/10 min).

The expected non-hydrolytic sol-gel reaction is described in Figure S2. It leads to isopropyl benzoate as a by-product. TDA-GC-MS coupling analyses were performed on the sample PS-Ti(O^i^Pr)_4_-BzA with the aim to confirm the proposed reaction mechanism thanks to the identification of the molecules contained in the system (especially the created by-product). The chromatogram with the identified molecules is displayed in Figure 6. The predominant molecule in PS-Ti(O^i^Pr)_4_-BzA nanocomposite was the expected isopropyl benzoate, at a retention time of 7.7 min. A small signal was also present at a retention time of about 2.3 min and corresponds to isopropanol, due to a marginal amount of titanium isopropoxide hydrolysis reactions. No signal attributable to benzoic anhydride was present on the chromatogram, suggesting a complete conversion for the first reaction step.

To confirm the complete conversion of benzoic anhydride, by-products were extracted from the polystyrene matrix by immersion of the nanocomposite grounded into powder in glacial acetic acid for 24 h under stirring. The purified nanocomposite was recovered by filtration and analyzed. FTIR analyses on PS-Ti(O^i^Pr)_4_-BzA sample before and after extraction allowed confirming the efficiency of the extraction (Figure S3). Total disappearance of signals corresponding to ester by-product (C = O stretching vibration of ester close to an aromatic group at 1712 cm^−1^, C-O stretching vibrations of ester at 1272 cm^−1^ and at 1090 cm^−1^) [32] was evidenced and confirmed the efficiency of the purification. Moreover, the absence of a signal at 1770 cm^−1^ confirms the complete reaction of benzoic anhydride.

Another way to ascertain the by-products extraction in PS-Ti(O^i^Pr)_4_-BzA nanocomposite was to perform thermogravimetric analyses. TGA thermograms before and after extraction are displayed in Figure S4. Before extraction, a mass loss of 14wt% was observed between 100 °C and 350 °C that could be assigned to isopropyl benzoate. For a complete condensation, the theoretical amount of isopropyl benzoate in composite was 29wt%. However, as the boiling point of this ester is 218 °C, i.e., lower than the reaction temperature, a part of the by-product could have evaporated during the nanocomposite elaboration in the internal mixer. Consequently, no conclusion on the condensation degree can be drawn from TGA results. After by-products extraction, a low mass loss of 2.6% still occurred before 350 °C, potentially due to condensation reactions occurring during the analysis. It was noteworthy that after the polystyrene degradation, 3.5wt% of inorganic residue was obtained in the nanocomposite before by-products extraction which is lower than the expected amount of inorganic fraction of 5wt%. Moreover, a part of the inorganic compound was also probably extracted from the nanocomposite during the immersion in glacial acetic acid. Indeed, the amount of inorganic residue at 525 °C (2.3wt%) is lower than that measured before extraction (3.5wt%).

As previously described, the condensation degree was determined by XPS analysis on purified sample, according to the respective area of the two signals at 530 eV and 532 eV (Figure S5). A condensation degree value of 26% was obtained, slightly higher than the condensation degree of 20% determined for TiO_2_ synthesized from acetic anhydride in the PS-Ti(O^i^Pr)_4_-Ac_2_O nanocomposite. Wang et al. [16] also obtained a low condensation degree for the synthesis of TiO_2_ fillers by the benzoic anhydride route in toluene. Indeed, a calcination yield of 53% was determined for this sample whereas the calcination yields for samples prepared by the ether and acetophenone routes were much higher (>90%).

**Figure 7:**
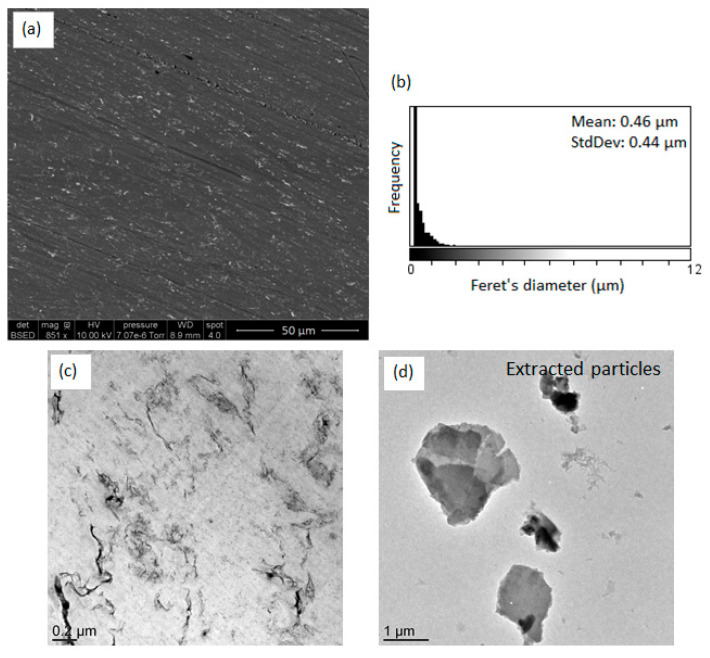
(**a**) SEM image of PS-Ti(O^i^Pr)_4_-BzA nanocomposite, (**b**) Feret’s diameter distribution of observed objects on the SEM micrograph, (**c**) TEM image of PS-Ti(O^i^Pr)_4_-BzA nanocomposite and (**d**) TEM image of extracted fillers from PS-Ti(O^i^Pr)_4_-BzA nanocomposite.

As elucidated in the first part of this study, the influence of the inorganic precursor/polymer affinity and of the condensation degree on the morphology was evidenced. The dispersion state of TiO_2_ fillers in PS-Ti(O^i^Pr)_4_-BzA sample was investigated by electron microscopy. SEM and TEM micrographs are displayed in Figure 7. The in situ synthesis of TiO_2_ from Ti(O^i^Pr)_4_ and benzoic anhydride led to the formation of nano-objects with a homogeneous dispersion according to SEM images. TEM observations of the nanocomposite revealed a platelet-like morphology, with fillers observed on the edge, which was also evidenced by the TEM image of extracted fillers. The thickness of fillers appeared lower than 50 nm on the TEM micrograph of PS-Ti(O^i^Pr)_4_-BzA nanocomposite, and their length was in the micrometer range. This filler shape was very different compared with PS-Ti(O^i^Pr)_4_-Ac_2_O sample. For the first time, we demonstrated the possibility to control a specific filler shape by the association of NHSG and reactive polymer processing. Actually, three factors had a significant influence on the morphology of obtained fillers: the nature of residual groups, the synthesis route, and the solvent [15]. The presence of phenyl groups in the case of PS-Ti(O^i^Pr)_4_-BzA sample could lead to the formation of π-π interactions between aromatic rings of polystyrene matrix and aromatic rings of benzoate groups which could influence the growth and shape of the TiO_2_ fillers.

As previously observed, TiO_2_ synthesized in situ by the benzoic anhydride route in our experimental conditions was amorphous, as shown by X-ray diffractograms in Figure S6. A broad signal was present at a low angle (at 5.01°) in the PS-Ti(O^i^Pr)_4_-BzA diffractogram. As seen for PP-Ti(O^i^Pr)_4_-Ac_2_O and PS-Ti(O^i^Pr)_4_-Ac_2_O nanocomposites, the presence of this signal may suggest a fillers organization at a long distance.

In the literature, another aromatic oxygen donor was used for the synthesis of TiO_2_ and BaTiO_3_ which is the acetophenone [16,33,34]. The reaction mechanism between Ti(O^i^Pr)_4_ and ketones was proposed by Garnweitner et al. [33] (Figure S7). This reaction involves the elimination of 1,3-diephenylbut-2-en-1-one (dypnone) and isopropanol. Another mechanism has been proposed by Pazik et al. [35] and leads to the formation of the ketal compound PhCMe(O^i^Pr)_2_ considering two equivalent of acetophenone in relation to Ti atoms.

**Figure 8:**
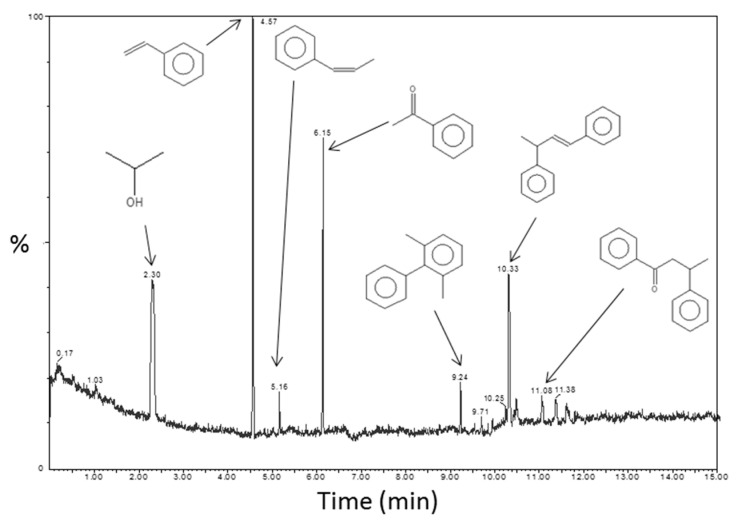
Chromatogram of PS-Ti(O^i^Pr)_4_-Aph nanocomposite with signals identification obtained by TDA-GC-MS coupling analyses (200 °C/10 min).

TDA-GC-MS coupling analyses were performed on the PS-Ti(O^i^Pr)_4_-Aph nanocomposite for confirming the proposed reaction mechanism. A chromatogram with corresponding molecules identified by mass spectrometry is displayed in Figure 8. The first signal at 2.3 min corresponding to isopropanol was one of the expected by-products of the reaction mechanism described in Figure S7. The highest signal was attributable to styrene and can be the result of residual monomers from the polystyrene matrix or a reductive dehydration of acetophenone with isopropanol [36]. A small amount of styrene was actually present in the chromatogram of the PS matrix (not shown) but the presence of styrene can be explained by the contribution of the two phenomena mentioned above. The presence of a signal for prop-1-en-1-ylbenzene at 5.2 min can be explained in the same way. A part of acetophenone did not react, as evidenced by the presence of the signal at 6.2 min. Neither dypnone nor PhCMe(O^i^Pr)_2_ were present in the PS-Ti(O^i^Pr)_4_-Aph composite according to this analysis. Indeed, the formation of dypnone would involve the presence of a signal at around 12.3 min in the chromatogram. However, some other molecules with two aromatic groups were identified: 1,3-dimethyl-2-phenylbenzene, 3-phenylbut-1-en-1-ylbenzene and 1,3-diphenylpropan-1-one. These molecules could result from reactions between intermediate reactants and by-products. The reaction mechanism was not actually as simple as described previously by Garnweitner et al. [33].

The residual reactants and by-products were extracted from the polystyrene matrix by immersion of nanocomposites grounded into powder in glacial acetic acid for 24 h under stirring, as described for PS-Ti(O^i^Pr)_4_-BzA sample. TGA analyses before and after extraction were performed (Figure S8) to confirm the total elimination of residual acetophenone as well as other by-products.

Before extraction, the mass loss between 100 °C and 350 °C was around 7.3wt%. As seen by TDA-GC-MS coupling analyses, this mass loss corresponded to residual acetophenone and by-products. The expected amount of by-product for a complete condensation was around 16wt% but the presence of unreacted oxygen donor and the possible evaporation of isopropanol and dypnone during the synthesis prevented the determination of a condensation degree. The residual inorganic part at 525 °C was also lower than expected (3.2wt% instead of 5.0wt%). The immersion of nanocomposite in glacial acetic acid allowed extracting acetophenone and by-products (2.1wt% of mass loss between 100 °C and 350 °C instead of 7.3wt%). Besides a lower inorganic residue measured at high temperature after purification may be as in the case of PS-Ti(OiPr)_4_-BzA nanocomposite due to a partial extraction of low condensed titanium-based species from the nanocomposite. These analyses confirmed the uncomplete reaction of the acetophenone contrary to the previously used benzoic anhydride.

All further following characterizations were performed on purified PS-Ti(O^i^Pr)_4_-Aph sample, i.e., after extraction step of by-products by immersion in glacial acetic acid.

**Figure 9:**
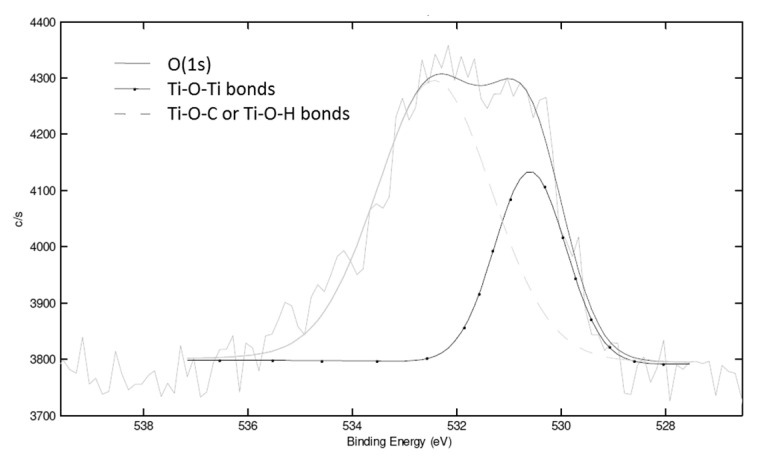
Deconvolution of the XPS O(1s) peak of the PS-Ti(O^i^Pr)_4_-Aph nanocomposite.

XPS analysis was used to determine the condensation degree of the NHSG reaction. From the C, O and Ti atomic concentrations of 97.5, 1.1 and 0.3%, respectively, and from the area of the O(1s) signal at around 530 eV and 532 eV (-Ti-O-Ti- and -Ti-O-H or -Ti-O-C respectively) (Figure 9), a condensation degree of 45% was determined which was higher than for PS-Ti(O^i^Pr)_4_-BzA sample (26%).

**Figure 10:**
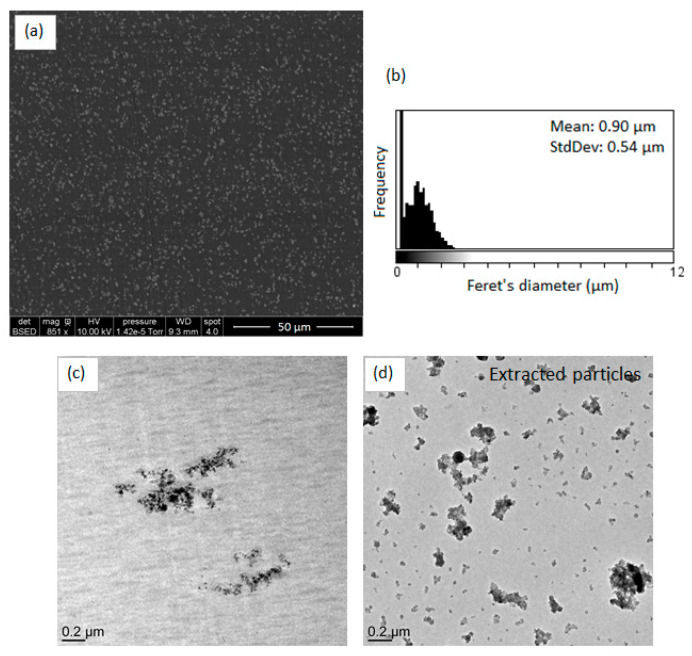
(**a**) SEM image of PS-Ti(O^i^Pr)_4_-Aph nanocomposite, (**b**) Feret’s diameter distribution of observed objects on the SEM micrograph, (**c**) TEM image of PS-Ti(O^i^Pr)_4_-Aph nanocomposite and (**d**) TEM image of extracted fillers from PS-Ti(O^i^Pr)_4_-Aph nanocomposite.

SEM and TEM micrographs of the PS-Ti(O^i^Pr)_4_-Aph nanocomposite and extracted fillers are displayed in Figure 10. We can observe that the use of acetophenone as an oxygen donor led to the formation of TiO_2_ nanofillers forming homogeneously dispersed aggregates with a mean diameter of approximately 900 nm. The morphology and the organization of TiO_2_ fillers in the PS matrix were close to the ones observed for PS-Ti(O^i^Pr)_4_-Ac_2_O composite. The TEM micrograph of extracted fillers (Figure 10d) showed the presence of single fillers and aggregates. The mean Feret’s diameter of primary fillers, determined by ImageJ software, was 15.0 nm ± 4.2 nm. Although the oxygen donor contains an aromatic group, the TiO_2_ fillers morphology is quite different from the TiO_2_ fillers synthesized from benzoic anhydride evidencing again also the role of the condensation degree on the final morphology.

As shown by X-ray diffractogram at wide angle (Figure S9), TiO_2_ synthesized in situ in viscous polystyrene is amorphous. A slight increase of the intensity was observed in the PS-Ti(O^i^Pr)_4_-Aph pattern at a low angle that could again suggest a better organization of fillers at a long distance as seen previously for the other PS based-systems.

As already described, oxygen donor played an essential role in the particles’ morphology and dispersion in non-hydrolytic sol-gel reactions in the solvent medium [16,37,38]. We depicted a similar effect in this work while the polystyrene acts as a solvent. In our case, the selection of the non-hydrolytic sol-gel route and the oxygen donor associated to the polymer processing conditions allowed tuning the particles’ morphology. We have underlined this potentiality and this richness by comparing two aromatic oxygen donors, leading to distinct morphologies.

## 4. Conclusions

The aim of this study was to demonstrate the possibility of controlling the shape of the TiO_2_ synthesized by associating the NHSG in a viscous polymer through a reactive polymer process. In that frame, two ways of synthesis were deeply scrutinized by either modifying the nature of the polymer or modulating the nature of the oxygen donor. First, the size and dispersion of TiO_2_ fillers synthesized in situ by non-hydrolytic sol-gel route in respectively PP and PS polymer matrices were compared. According to SEM, TEM, and XPS characterizations, TiO_2_ fillers in PP-based composite had a size around 1 µm and were well-condensed (~79%) whereas in PS-based composite, the nanofillers formed aggregates with a size around 200 nm associated to lower condensation degree (~20%). This difference was shown arising from the better affinity of the acetic anhydride with the PS matrix but also from the PS highest viscosity compared to the PP one.

Secondly, the influence of the oxygen donor nature (needed for NHSG route) on the size and morphology of TiO_2_ fillers was also evaluated in the case of the PS matrix. In that frame, PS/TiO_2_ nanocomposites synthesized from benzoic anhydride as the oxygen donor have a platelet-like filler. In the case of the PS-Ti(O^i^Pr)_4_-Aph nanocomposite, we obtained primary nanoparticles approximately 15 nm in diameter, assembled in micrometer scale aggregates. XPS analyses indicated a higher condensation degree for TiO_2_ fillers synthesized from acetophenone than from benzoic anhydride (45% and 26% respectively). It is worth noting that it is the first report that a specific shape of a filler can be obtained through a molten polymer process by controlling the affinity between the oxygen donor and the polymer.

These results on the non-hydrolytic sol-gel route transposed to viscous polymer medium conditions are very encouraging as they highlighted the impact of the nature of the polymer and of the oxygen donor on the morphology of the created TiO_2_. This approach could further be exploited to target polymer-based materials with specific properties.

## Figures and Tables

**Table 1:** Structure and selected properties of reactants.

	Titanium Isopropoxide	Acetic Anhydride	Benzoic Anhydride	Acetophenone
Chemical structure	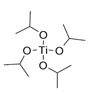	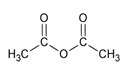	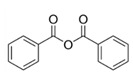	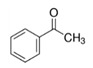
Molar mass (g.mol^−1^)	284.22	102.09	226.23	120.15
Melting point (°C)	14	−73	43	20
Boiling point (°C)	232	140	360	202

**Table 2:** Nanocomposite samples description.

Sample	Polymer Matrix	TiO_2_	Expected by-Product	Boiling Point of by-Product (°C)
PP-ref	Polypropylene	-	-	-
PS-ref	Polystyrene	-	-	-
PP/TiO_2_-ref	Polypropylene	TiO_2_ Aeroxide P25	-	-
PS/TiO_2_-ref	Polystyrene	TiO_2_ Aeroxide P25	-	-
PP-Ti(O^i^Pr)_4_-Ac_2_O	Polypropylene	Titanium isopropoxide + 2 eq Acetic anhydride	Isopropyl acetate	90
PS-Ti(O^i^Pr)_4_-Ac_2_O	Polystyrene	Titanium isopropoxide + 2 eq Acetic anhydride	Isopropyl acetate	90
PS-Ti(O^i^Pr)_4_-BzA	Polystyrene	Titanium isopropoxide + 2eq Benzoic anhydride	Isopropyl benzoate	218
PS-Ti(O^i^Pr)_4_-Aph	Polystyrene	Titanium isopropoxide + 4 eq Acetophenone	1,3-Diphenyl-2-buten-1-one/Isopropanol	395/83

**Table 3:** Solubility parameters calculated through Fedors’ method [17].

Compound	Polypropylene	Polystyrene	Acetic Anhydride
**δ (MPa) ^1/2^**	16.0	21.1	19.8

## Data Availability

Not applicable.

## Supplementary Materials

The following supporting information can be downloaded at: https://www.mdpi.com/article/10.3390/polym14112273/s1, Figure S1: TGA thermograms of (a) PP-ref and PP-based nanocomposites and (b) PS-ref and PS-based nanocomposites. Analyses were performed under helium atmosphere with a heating rate of 10 °C min^−1^; Figure S2: NHSG reaction scheme between titanium isopropoxide and benzoic anhydride; Figure S3: FTIR spectra of PS-Ti(O^i^Pr)_4_-BzA nanocomposite before and after by-products extraction by immersion in glacial acetic acid for 24 h; Figure S4: TGA thermograms of PS-Ti(O^i^Pr)_4_-BzA nanocomposite synthesized from Ti(O^i^Pr)_4_ and benzoic anhydride before and after by-products extraction by immersion in glacial acetic acid for 24 h; Figure S5: Deconvolution of the XPS O(1s) peak of the PS-Ti(O^i^Pr)_4_-BzA nanocomposite; Figure S6: X-ray diffractograms of PS-ref and PS-Ti(O^i^Pr)_4_-BzA nanocomposite; Figure S7: Proposed NHSG reaction scheme between titanium isopropoxide and acetophenone; Figure S8: TGA thermograms of PS-Ti(O^i^Pr)_4_-Aph nanocomposite synthesized from Ti(O^i^Pr)_4_ and acetophenone before and after by-products extraction by immersion in glacial acetic acid for 24 h; Figure S9: X-ray diffractograms of PS-ref and PS-Ti(O^i^Pr)_4_-Aph nanocomposites.
