# Supplementary material for: Tuning Polymer/TiO2 Nanocomposites Morphology by In Situ Non-Hydrolytic Sol-Gel Syntheses in Viscous Polymer Medium: Influence of the Polymer Nature and Oxygen Donor
Source: Polymers (Basel). 2022 Jun 2;14(11):2273. doi: 10.3390/polym14112273 (PMC9183041; doi:10.3390/polym14112273)
Supplement: Supplementary file 1 [file polymers-14-02273-s001.zip › polymers-1671312-supplementary.pdf]

Supporting information

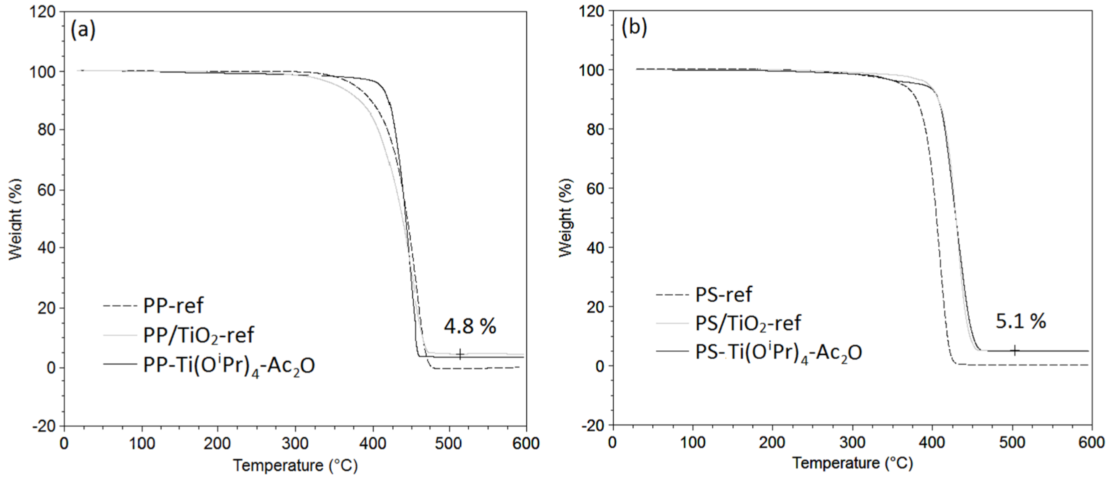

Figure S1 - TGA thermograms of (a) PP-ref and PP-based nanocomposites and (b) PS-ref and PS-based nanocomposites.

Analyses were performed under helium atmosphere with a heating rate of 10 °C min^{-1}

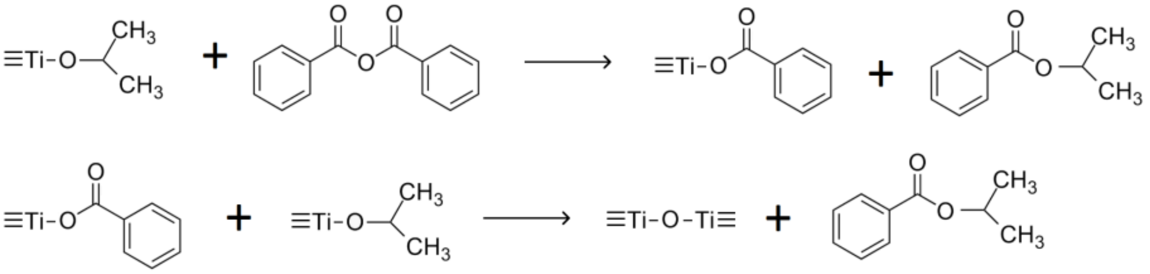

Figure S2- NHSG reaction scheme between titanium isopropoxide and benzoic anhydride

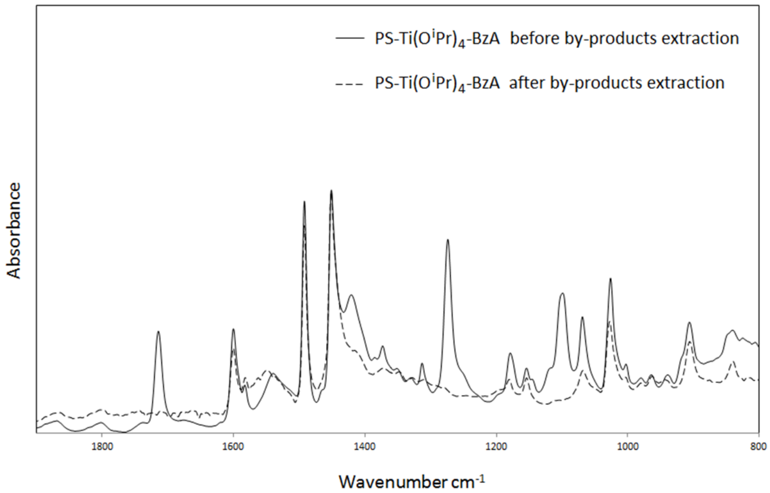

Figure S3 - FTIR spectra of PS-Ti(O^{i}Pr)$_4$-BzA nanocomposite before and after by-products extraction by immersion in glacial acetic acid for 24 h

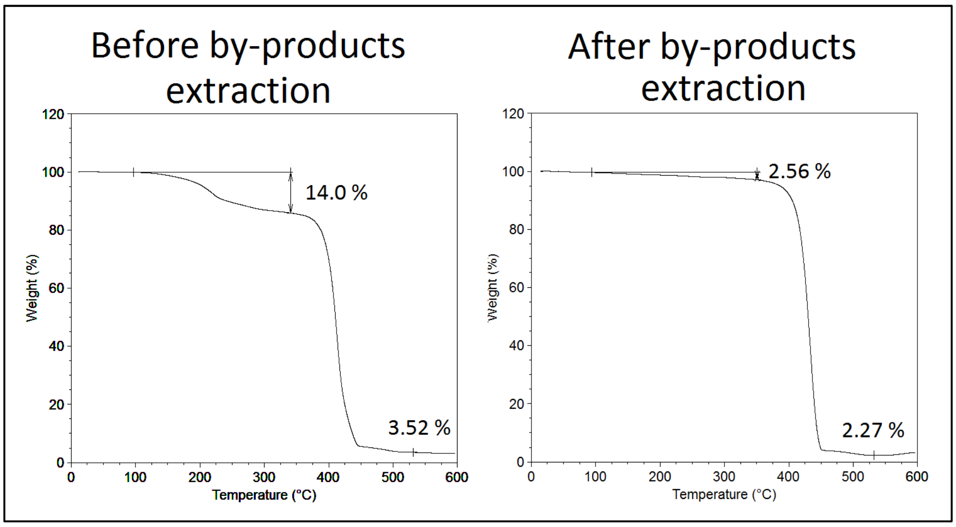

Figure S4 - TGA thermograms of PS-Ti(O^{i}Pr)$_4$-BzA nanocomposite synthesized from Ti(O^{i}Pr)$_4$ and benzoic anhydride before and after by-products extraction by immersion in glacial acetic acid for 24 h

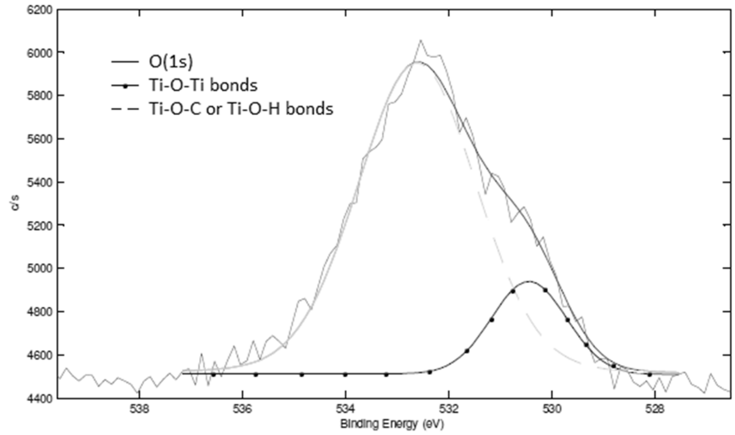

Figure S5 - Deconvolution of the XPS O(1s) peak of the PS-Ti(O^{i}Pr)$_4$-BzA nanocomposite

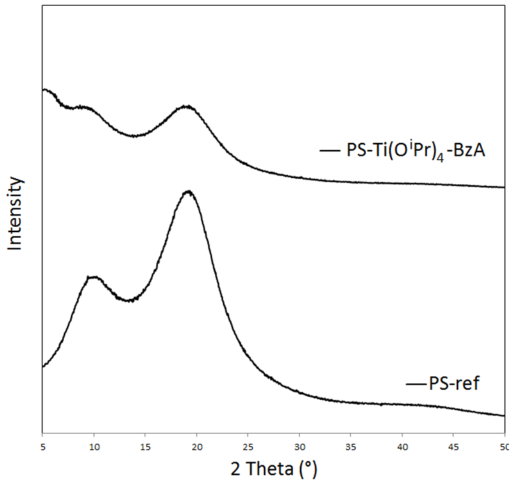

Figure S6 - X-ray diffractograms of PS-ref and PS-Ti(O^{i}Pr)$_4$-BzA nanocomposite

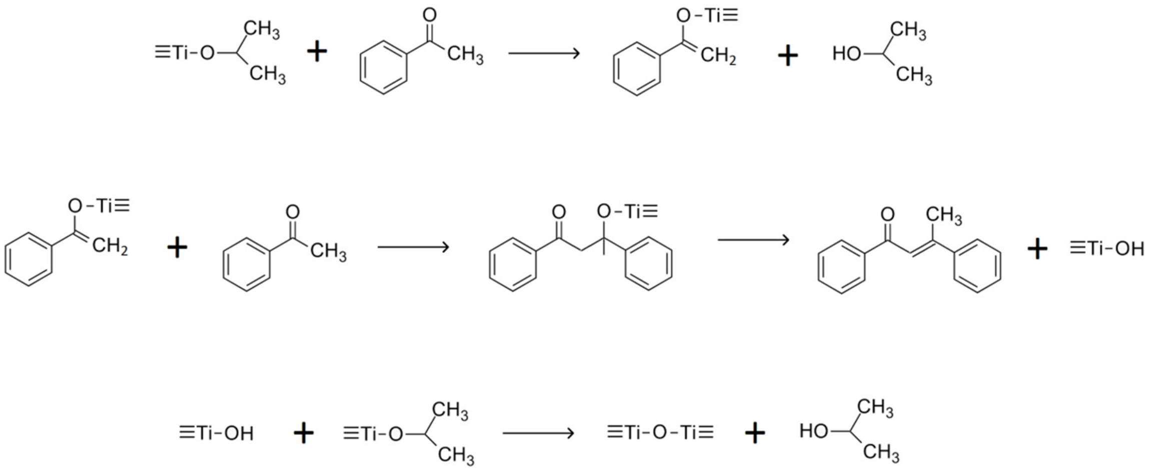

Figure S7 - Proposed NHSG reaction scheme between titanium isopropoxide and acetophenone

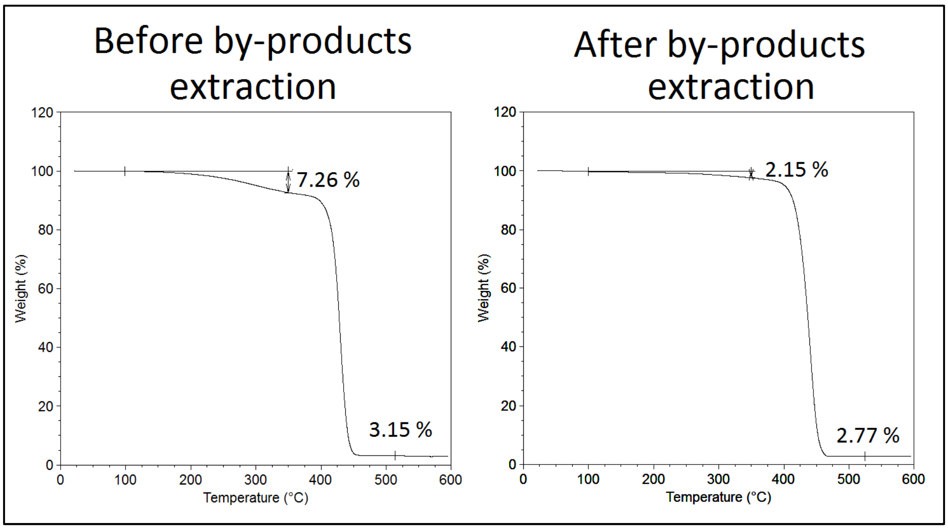

Figure S8 - TGA thermograms of PS-Ti(O^{i}Pr)$_4$-Aph nanocomposite synthesized from Ti(O^{i}Pr)$_4$ and acetophenone before and after by-products extraction by immersion in glacial acetic acid for 24 h

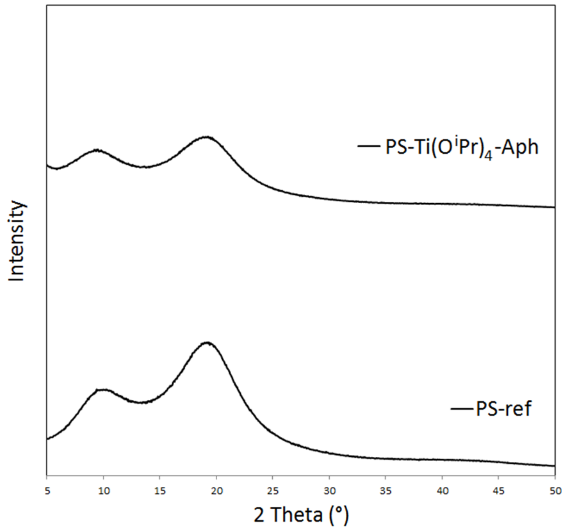

Figure S9 - X-ray diffractograms of PS-ref and PS-Ti(O^{i}Pr)$_4$-Aph nanocomposites
